# Viability of diagnostic decision support for antenatal care in rural settings: findings from the Bliss4Midwives Intervention in Northern Ghana

**DOI:** 10.7189/jogh.09.010420

**Published:** 2019-06

**Authors:** Ibukun-Oluwa Omolade Abejirinde, Vincent De Brouwere, Jos van Roosmalen, Maurits van der Heiden, Norbert Apentibadek, Azucena Bardají, Marjolein Zweekhorst

**Affiliations:** 1 Athena Institute, Vrije Universiteit, Amsterdam, the Netherlands; 2 Institute of Tropical Medicine, Maternal and Reproductive Health Unit, Department of Public Health, Antwerp, Belgium; 3 ISGlobal, Hospital Clínic – Universitat de Barcelona, Barcelona, Spain; 4 Leiden University Medical Centre, Department of Obstetrics, the Netherlands; 5 the Netherlands Organisation for Applied Scientific Research (TNO), Delft, the Netherlands; 6 Association of Church Development Projects (ACDEP), Northern Ghana

## Abstract

**Background:**

Antenatal screening is useful for early identification and management of high-risk pregnancies. In low-resource settings, provision of the full complement of tests is limited and diagnostic referrals incure additional costs for pregnant women. We assessed the viability of Bliss4Midwives (B4M) - a point-of-care diagnostic decision support device for decentralized screening of pre-eclampsia, gestational diabetes and anaemia during antenatal care (ANC).

**Methods:**

The device was piloted in seven health facilities across two districts in Northern Ghana over a ten-month period. Health workers were expected to screen women at each ANC visit till delivery. All screening records from the device were automatically archived digitally and later downloaded. After removing duplicates or invalid entries, descriptive quantitative analysis was carried out with IBM SPSS Statistics (version 23). B4M usage behavior, diagnostic and referral outcome were analyzed.

**Results:**

Health workers conducted 1323 partial or full antenatal screening on 940 women, resulting in decision support for 835 (88.8%) B4M beneficiaries. Diagnostic referral was eliminated for 708 (84.7%) beneficiaries, with 335 (40.1%) of these from facilities without on-site diagnostic alternatives. Of visits with complete data, 92/559 (16.4%) women were screened in their first trimester, 28/940 (2.9%) had 4+ B4M visits and 107/835 (12.8%) women were recommended for urgent referral to a higher-level facility on the first visit. Follow-up screenings flagged an additional 17 women for urgent referral with 10 cases of repeated alerts in five women. Wide variations between high (9 months use) and low adopting (1.5 months use) facilities were observed, with some similarities in usage trend.

**Conclusions:**

B4M helped decentralize ANC screening and decrease unnecessary referrals. Project outcomes were influenced by implementation strategy, technical features and behavioural dispositions of users and beneficiaries.

Alongside counselling during antenatal care (ANC), screening for anaemia, hyperglycemia and sexually transmitted infections, amongst others, is important for the management of pregnancy-related complications [1,2]. In rural settings, laboratories may be unavailable or ill equipped such that pregnant women cannot receive the full complement of screening at the point-of-care (POC). They are therefore subject to diagnostic referral and may have to travel long distances, which is a barrier to accessing the optimal package of maternal health services [3]. Additionally, front-line workers are not always trained or equipped to properly conduct ANC screening. Other factors that contribute to diagnostic gaps at peripheral levels include staff shortages, geographic isolation and cost implications [4]. These pose a challenge for ensuring early identification and timely decision-making on the management of conditions that could endanger the health of mother and child [5].

In Ghana, up to 35% of pregnant women were anaemic (haemoglobin <11g/dl) at their first ANC visit, with severe anaemia and malaria accounting for 26% of maternal mortality [6]. Independent cross-sectional studies in a Ghanaian tertiary hospital indicate about 10% prevalence of gestational diabetes and 7.5% of pre-eclampsia in pregnant women [7,8]. Although ANC coverage has generally improved in Ghana, and over 80% of pregnant women had at least one contact with a skilled health provider in 2016, recent data shows disparities between its 10 regions (100% ANC coverage in the Northern region (NR) but only 70% in the Volta region) [6]. Evidence also indicates that the recommend number and timing of visits are not being achieved. Nationally, 76% of women attend at least four visits and less than 50% initiate ANC within the first trimester, with rural women being less likely to engage with the formal health sector [6,9]. Only 83% of women in rural areas have 4+ ANC visits compared to urban women (92%) [10]. In the Northern and Upper East regions- two regions with the highest poverty levels, 4+ ANC visits were 73% and 93% respectively [10,11]. While efforts have been made to ensure that women in remote areas can access maternal and child services, not all health facilities have the capacity for diagnostic screening. Women thus have to travel to alternative sites for basic tests, increasing the financial burden for transport and service charges. Poor feedback between facilities results in low referral compliance, affecting patient monitoring and accurate record keeping [6]. To ensure access to quality care for all pregnant women, there is a need for alternative strategies that consider contextual limitations.

Point-of-care and decision support technologies are reported to improve service delivery and aid task shifting, bridge the know-do gap and empower workers to take clinical decisions [12-14]. In June 2016, a consortium of five Dutch organizations and two Ghanaian partners launched an integrated diagnostic and decision support device in two districts of Northern Ghana. The one-year proof-of-concept tagged Bliss4Midwives (B4M) aimed to reduce pregnancy-related complications by improving quality antenatal care (ANC) through non-invasive diagnostic tests supported by decision algorithms. By facilitating POC screening for pre-eclampsia, gestational diabetes and anaemia, the intervention was expected to facilitate early detection, woman-friendly consultation and streamlined referral pathways.

To assess its viability, the pilot project was evaluated. We present an overview of the intervention alongside findings on usage behaviour and referral recommendations. Analysis focused on how each health facility performed with using B4M. Only records of women who were screened with the device were analyzed, regardless of the total number of women that attended ANC visits during the project period. Pregnant women’s perceptions of the device and its influence on women-provider relationships have been reported elsewhere [15].

## METHODS

### The B4M device

**Figure 1:**
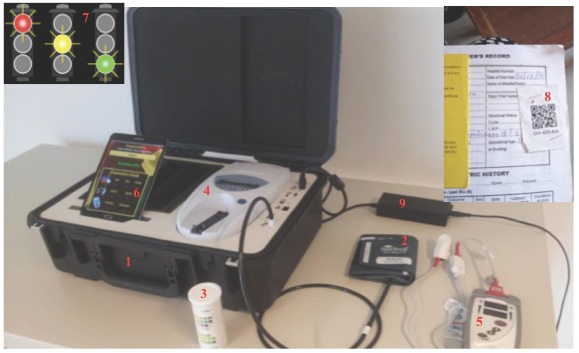
B4M device. 1 – Portable water and heat resistant dustproof case; 2 – Automated blood pressure cuff; 3 – Urinary glucose and protein Chemistrips; 4 – Urisys 1100® Urine Analyzer; 5 – Pronto-7® Rainbow Pulse CO-Oximetry device; 6 – Android tablet with decision support algorithms; 7 – Traffic-signaling alert system; 8 – Unique QR code for easy tracking and recall of patient records; 9 – AC adapter for charging the device.

Seven prototype B4M devices were assembled (**Figure 1**). Each device is composed of three diagnostic and two supportive components.

#### B4M diagnostic components

- Non-invasive Pronto-7® Rainbow Pulse CO-Oximetry™ device (Masimo Corporation, Irvine, California, USA) for arterial oxygen haemoglobin, pulse rate and total haemoglobin concentration. Signals are transmitted from the woman to the device through infrared sensors mounted on a finger clip.
- Suntech Medical® Advantage™ OEM non-invasive blood pressure (OEM-NIBP) device with automated arm cuff (Suntech Med Inc, Morrisville, NC, USA).
- Urisys 1100® Urine Analyzer (Roche Diagnostics, Risch-Rotkreuz, Switzerland), which automatically reads protein and glucose levels in urine through Chemstrip®. It operates on the principle of reflectance photometry, eliminating the need for visual interpretation of results, which is prone to error.

Diagnostic components meet requirements of the Food and Drug Administration or European Standard of Electronic Engineering and conform to standards for developing medical prototypes- ISO 13485 [16].

#### B4M supportive components

- An android tablet that paired with diagnostic components via Bluetooth was pre-programmed with decision support algorithms according to Ghana Health Service guidelines. The decision support component used a traffic-light system to indicate level of risk or referral urgency while prompting patient counseling, management or referral. Textual recommendations accompanied visual indicators on whether hospital referral was needed. Where necessary, immediate actions to take were indicated. Screening records were saved without Internet access and program managers regularly exported the data to an external device.
- Pregnant women were linked to the system on the first B4M screening visit via Quick Response (QR) codes affixed to their health record booklets. This allowed quick and easy recall of records on subsequent visits.

Diagnostic data were automatically or manually (only hemoglobin) uploaded to the tablet. In addition to updating clinical history and hemoglobin (Hb) results manually, B4M users were expected to input delivery date and outcome as well as summarized notes on their observations or actions. Devices could be locked and transported between locations. Except for the haemoglobinometer that used disposable batteries, a rechargeable lithium battery powered all prototype components. When fully charged, components could operate without electricity for up to seven hours. Consumables (Chemstrip®, disposable batteries, finger sensors) were replenished by the project on request. Other technical details of the device are beyond the scope of this paper.

### Implementation setting and process

The device was implemented in the Upper East Region (UER) and Northern Region (NR) of Ghana. All intervention sites are predominantly rural and about half of the population is illiterate [17-19]. Five health facilities per region were enrolled, but technical and implementation challenges such as defective devices and transfer of trained staff reduced the total number to seven- four health facilities (facilities A-D) from UER and three facilities (facilities E-G) from NR. Because it was a pilot, B4M was used in addition to the pre-existing paper-based ANC routine and health workers were expected to screen all women who came for ANC at each visit till delivery.

Three devices were situated in each region and one device reserved for backup. Facility selection was largely guided by high ANC-load per facility, remote distance from referral hospital and possibility that more women would benefit from the device. Devices were assigned to facilities on a fixed or rotational basis. Higher caseload facilities such as hospitals had a fixed device. Where a facility was too remote for convenient rotation (eg, facility G), it had a fixed device. Each B4M visit ideally involved a systematic step-wise process starting with enrolment of first time beneficiaries using their data and history, followed by presenting complaints, diagnostic screening and referral decision. Client counselling concluded each visit. Based on pre-intervention estimates, 100 pregnant women per health facility were expected to be screened in a year, with 40 women completing at least four ANC visits between early pregnancy and delivery [16].

Twenty-five midwives and community health workers received two-days training on B4M. This included refresher training on managing pregnancy complications and principles of quality ANC. Three field personnel with technical backgrounds, and two program officers (one in each region) were also trained to provide technical support.

### Data collection and analysis

The Android tablet was programmed to automatically archive all screening records and usage information in a downloadable repository. Such information included: kit number, B4M visit date, B4M visit number and QR code, mother’s name, parity, expected date of delivery, gestational age, delivery date and outcome of delivery, patient history, presenting complaints, diagnostic decisions, action (ie, counseling, testing and treatment) and referral recommendations. The study sample was limited to all women who were screened with the device; therefore excluding those who attended ANC visits during the project period without being screened with B4M. A member of the project team downloaded the repository from each device. Records were de-identified, cleaned and sorted for duplicates or invalid entries (eg, records from training or trial sessions) using Excel. Descriptive quantitative analysis of records archived over a 10-month period (15^th^ June 2016 to 18^th^ April 2017) was carried out using IBM SPSS Statistics (version 23) (IBM Inc, Armonk, NY, USA).

### Ethical considerations

Navrongo Health Research Centre Institutional Review Board (Approval ID: NHRCIRB18) and EMGO+ Scientific Committee of the Amsterdam Public Health Institute (Reference Number: WC2017-026), granted study approval. Most information collected in the B4M repository represented data that should also be recorded in the paper-based maternal health record books at each facility. This includes the name, address, parity, age and gestational age of women. Because record books are not necessarily stored securely, they pose a higher privacy risk as anyone could easily access information on ANC attendees. To ensure data protection and privacy, the personal data of women were linked through the anonymised QR code, each with a unique alphanumeric project label (see **Figure 1**, item 8). Furthermore, each B4M user was assigned a username and password and only personnel with access could assign or scan QR codes, view personal and clinical data and conduct the tests with the device. Prior to assigning QR codes to ANC attendees at their first B4M screening, health workers were trained to enrol pregnant women into the study using a structured information sheet that explained the study procedure, benefits, risks and confidentiality. Women confirmed consent with a signature or thumbprint. The device was introduced as an additional station in the existing ANC workflow and could be locked and securely stored when not in use.

## RESULTS

### Beneficiary characteristics and B4M service delivery

In the seven intervention facilities, 1323 antenatal screenings were conducted in 940 women. With an average of three and a maximum of ten pregnancies per woman, 283 (30%) B4M beneficiaries were primigravidae (first pregnancy) and 78 (8%) were grand multiparous (>5 pregnancies). Up to 12% of beneficiaries represented high-risk age groups for pregnancy- 51 (5.4%) women were less than 18 years old, while 64 (6.8%) women were older than 35 years.

Actual device adoption over a ten-month period differed between regions and facilities, ranging from 1.5 to 9 months. Length of actual use was calculated as the duration between the first and last B4M screening record at each facility (**Table 1**). Health facilities in UER demonstrated higher usage behavior- accounting for 950 (71.8%) screening records and 646 (68.7%) beneficiaries. In the NR, 373 (28.1%) screening records represented B4M visits for 294 (31.2%) women.

**Table 1:** B4M service delivery

Health Facility*	Type of health facility†	Fixed or rotating device	Length of use (months)	Screening records N = 1323 (100%)	Women screened N = 940 (100%)
Upper East Region:					
Facility A	Secondary	Fixed	9	284 (21.5%)	252 (26.8%)
Facility B	Primary	Rotating (with facility C)	6	69 (5.2%)	63 (6.7%)
Facility C	Primary	Rotating (with facility B)	3.5	129 (9.8%)	78 (8.3%)
Facility D	Primary	Fixed	9	468 (35.4%)	253 (26.9%)
Northern Region:					
Facility E	Primary	Fixed	1.5	25 (1.9%)	25 (2.7%)
Facility F	Primary	Fixed	1.5‡	337 (25.5%)	258 (27.4%)
Facility G	Primary	Fixed	1.5	11 (0.8%)	11 (1.2%)

*Facility A is the ANC unit of a district hospital, which is the first level referral point for facilities B, C and D, which are health centers. Facility E is an independent public health unit of a district hospital, while F and G are health centers.

†Differentiated by level of service provision: primary facilities provide basic community-level services while secondary facilities are usually district hospitals with capacity for more specialized care.

‡Dating errors in 302 records.

### Compliance to B4M

**Figure 2:**
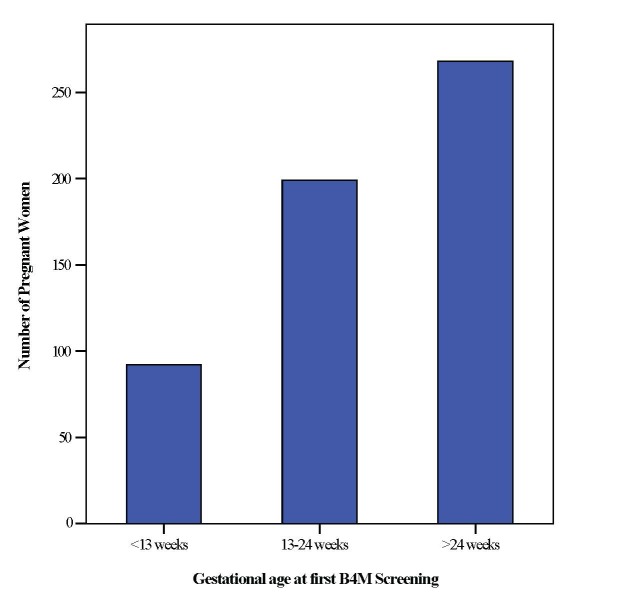
Gestational age at first B4M visit.

Due to missing values, it was only possible to calculate first B4M visit as a function of gestational age for 559 (59%) women (**Figure 2**). Of these, the number of women screened with B4M in their first trimester (<13weeks) was rather low – 92 (16.4%). Interestingly, a large number – 268 (47.9%) women were screened for the first time after 24 weeks gestation.

**Figure 3:**
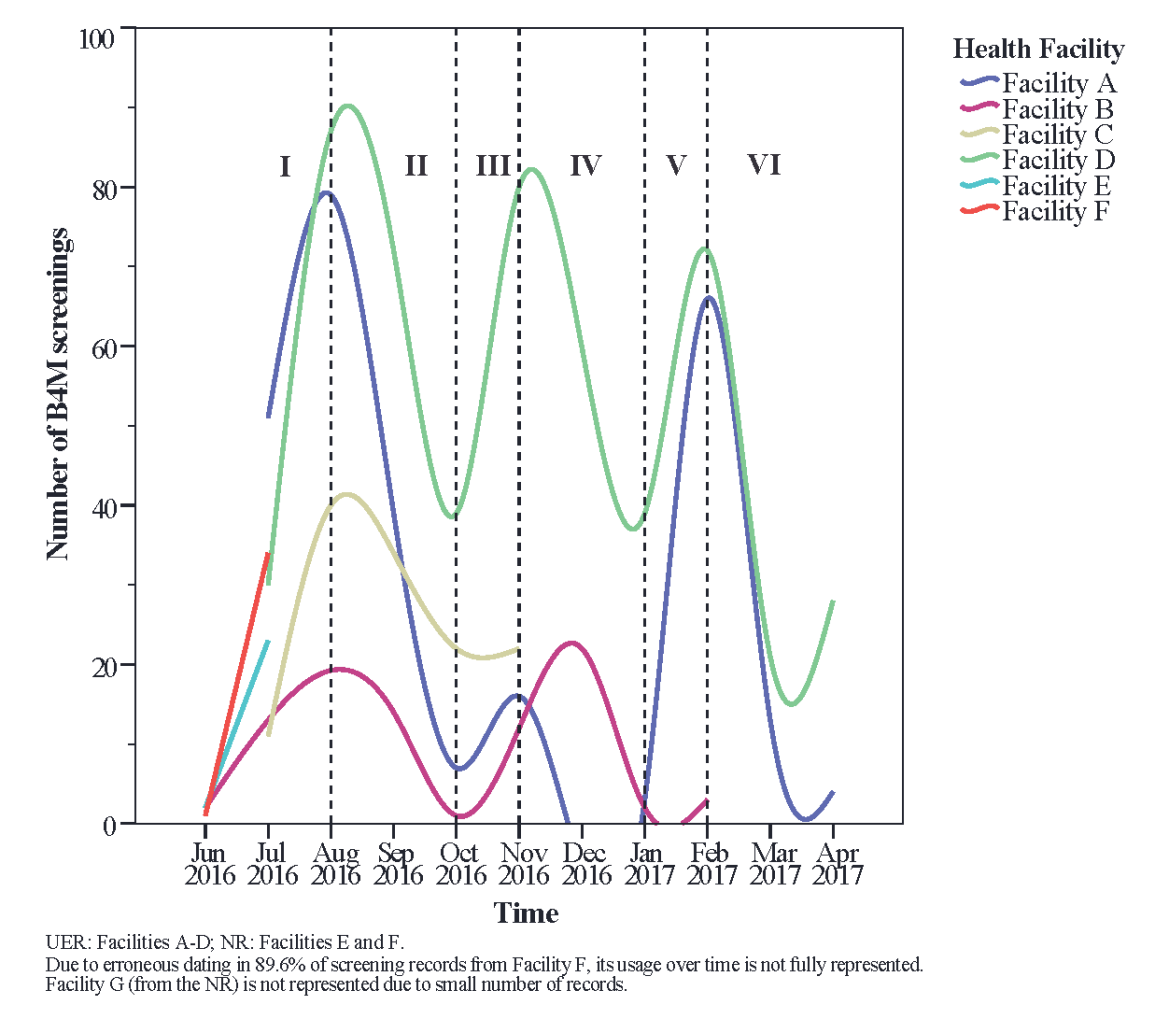
Trend analysis of B4M usage.

Usage trend over the project period was plotted to understand critical points of use or non-use of B4M. An unexpected wavelike pattern starting with a steady increase in number of screenings was found in all health facilities (**Figure 3**, phase I). Between August and October 2016 (phase II), number of records fell despite expectations that enrolled women will be re-assessed in repeat ANC visits, with new registrants supplementing these numbers. Facility A showed the widest decline at this stage, and did not use the device between December 2016 and January 2017 (phase IV). Despite phase IV cessation of B4M use in facility A and declined use in facility D, both sites manifested usage surge by February 2017 (phase V). With a slight exception in facility B, no facility reattained initial peak usage rates.

Irrespective of differences in facility type and implementation strategy, health facilities (more specifically A, B and D) seemed to follow a similar wave pattern from phases I through VI, with usage increasing and declining within the same time period.

**Figure 4:**
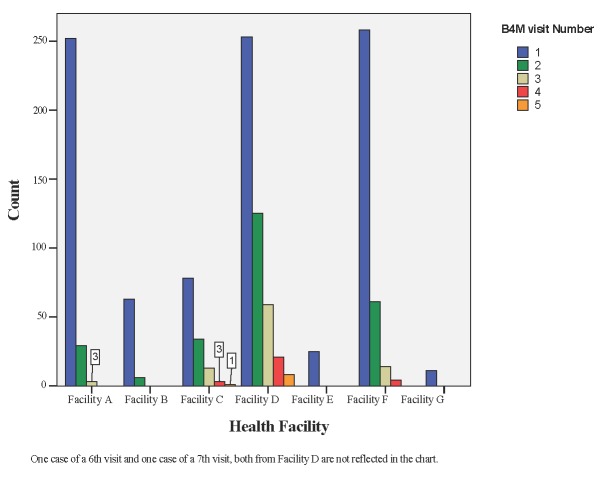
Compliance to repeat screening.

Of the 940 B4M beneficiaries, 28 (2.9%) received four or more screening visits. Although facilities A, D and F served about the same number of pregnant women, only facility D had the highest number of follow-up B4M screenings per woman- up to 8 (0.8%) women had 5+ B4M screening visits (**Figure 4**).

Information requiring manual entry was often not entered or had errors due to oversight on the part of users. Once detected, the monitoring team attempted to address this by encouraging users to pay attention to accuracy and completeness of entries. Nevertheless, manual feedback notes on health workers’ actions were entered in only half of the visits. Delivery outcome was entered in only 30 cases, although 536 women were due for delivery within the project timeline.

### Diagnostic results

#### Haemoglobin

**Figure 5:**
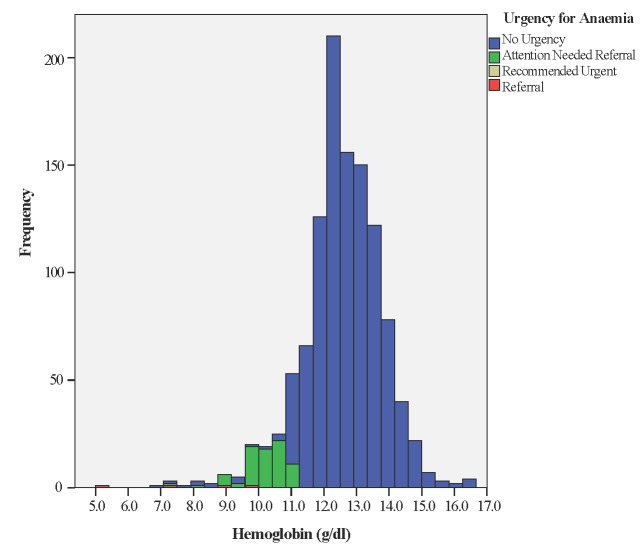
Screening for anaemia.

Unlike other test results, which were automatically uploaded to the Android tablet, Haemoglobin (Hb) results had to be entered manually by health workers. Of 1323 screening visits, 34 (2.5%) Hb measurements had typing errors (eg, missing the decimal point) and 78 (5.8%) did not include Hb data, leaving 1211 (91.5%) valid records. Mean Hb reading was 12.5 g/dl (SD = 1.3) with median 12.6 g/dl and range from 5.0 to 16.6 g/dl. Of valid records, 114 (9.4%) were between 7 and 11 g/dl, prompting recommendation of a 2-week follow-up visit and a reminder to give iron tablets and counsel on adequate pregnancy nutrition. Only one woman had Hb <7 g/dl, mandating urgent referral (**Figure 5**).

#### Blood pressure

**Figure 6:**
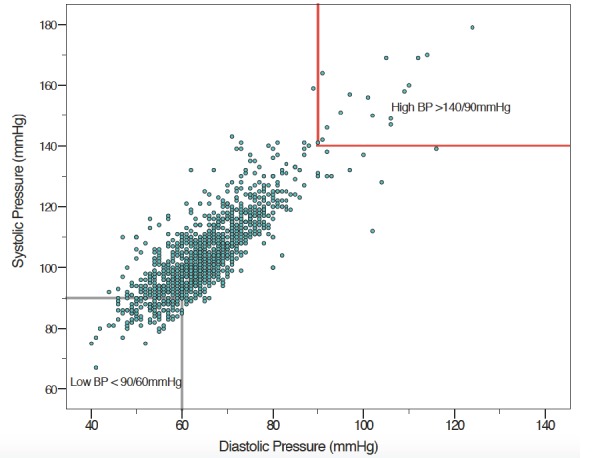
Blood pressure screening.

B4M screening records from 1287 (97.4%) visits included blood pressure (BP) measurements. Mean systolic pressure was 104.24 mm Hg (SD = 13.6) and mean diastolic 65 mm Hg (SD = 10.1), with median 102 mm Hg (range 67 to 191 mm Hg) and 64 mm Hg (range from 40 to 149 mm Hg), respectively (**Figure 6**).

#### Urinalysis (protein and glucose)

In 1315 (99.4%) of B4M-ANC visits, urinalysis had been conducted. In 115 (8.7%) of completed tests, an increased urine glucose (≥100 mg/dL) was identified, and in four instances, levels were >1000 mg/dl. Health workers were advised to refer women, based on increased urinary glucose following 28 visits. Scheduling a two-week follow-up screening was recommended in 83 instances. Urinary protein was elevated (≥30 mg/dL) in 164 (12.5%) samples. In eight cases, levels were as high as 500 mg/dl and workers were prompted to follow-up with testing for urinary tract infections. Results from blood pressure and urinalysis were collectively factored into the decision algorithm for action on pre-eclampsia; nine women were flagged for urgent referral and 117 women for close monitoring (ie, two-week follow-up).

### Decision support and referral advice

Combined analysis of the different diagnostic tests, woman’s history, presenting complaints and expected date of delivery guided B4M referral advice. We excluded 126 (10%) records with incomplete data on blood pressure, urinalysis or Hb, leaving 1197 (90%) screening records representing 835 women. If the recommendation was “no urgency” or “attention needed” health workers scheduled women for the next ANC visit in four or two weeks respectively. In decisions marked “urgent referral” or “direct action + urgent referral” (**Table 2**), women were expected to be referred to the next level of care immediately. Where the B4M decision was “referral recommended”, health workers were expected to decide a course of action using their clinical judgement and experience.

**Table 2:** Distribution of B4M referral advice

	No urgency	Attention needed	Referral recommended	Urgent referral	Direct action + urgent referral
Facility A	134 women	72 women	4 women	40 women	–
Facility B	17 women	38 women	3 women	1 woman	–
Facility C	35 women	19 women	1 woman	22 women	–
Facility D	143 women	74 women	1 woman	34 women	–
Facility E	9 women	6 women	2 women	5 women	1 woman
Facility F	113 women	39 women	9 women	3 women	–
Facility G	5 women	4 women	-	1 woman	–
**N = 835 (100%)**	456 women (54.6%)	252 women (30.1%)	20 women (2.3%)	106 women (12.6%)	1 woman (0.1%)

**Figure 7:**
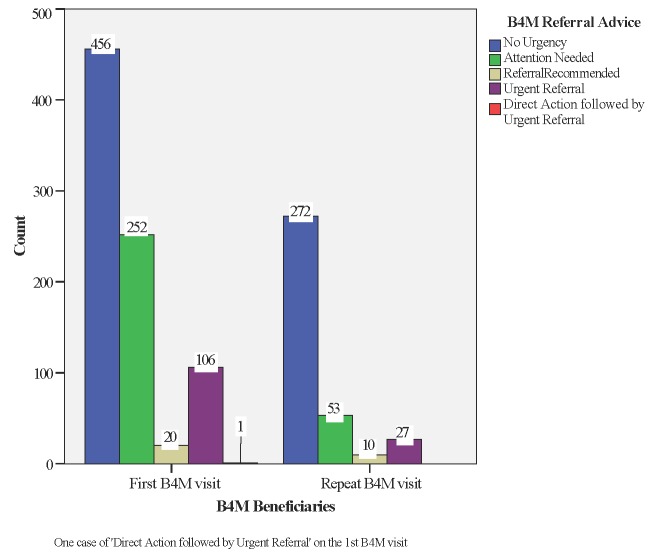
B4M referral advice per beneficiary.

Decision outcome for 107 (12.8%) of women with complete screening data at their first B4M visit was ‘urgent referral’ to a higher-level facility (**Figure 7**). In other cases, referral urgency was detected during follow-up visits, representing newly detected danger signs for 17 women (15 on the 2nd visit and two at the 3rd) and 10 instances (5 women) of repeat referral recommendation from a previous visit.

At the time of data collection, Facilities A and F both had functional laboratories where urinalysis and Hb testing could be done, while Facility E regularly used POC tests acquired through a different project. In other facilities, B4M was the primary “mobile laboratory”, in the absence of which diagnostic referral was common. **Table 2** shows that 708 (84.7%) women who received a B4M decision had no referral urgency (“no urgency” + “attention needed”). Of these, 335 (40%) women were tested in facilities without on-site diagnostic alternatives.

## DISCUSSION

By providing integrated diagnostic services at the point-of-care to almost 1000 women, the Bliss4Midwives pilot demonstrated the extent to which technology-driven alternatives for early detection and referral of selected conditions of pregnancy in rural settings are feasible. Even in the presence of alternatives, both primary and secondary facilities used the device, albeit to varying extents. Alongside individual, organisational and contextual factors, use of B4M can be linked to perceptions of its usefulness [20]. However, the creation of parallel workflows that strain workers and lead to adoption resistance could have influenced users compliance to the device [12,21,22]. Because service providers require interventions to be sensitive to their context and easily integrate into workflow [23,24], mobile Health (mHealth) usage can be low despite high perceived usefulness [25]. In light of contextual limitations such as high caseload and low staffing, and because using the device took longer, not all ANC attendees were screened with B4M [15]. Some health workers did not use all three screening components per visit, resulting in partial screening.

We found wide variations between high adopting and low adopting facilities with some pattern similarities. Higher usage behavior in the UER was partly explained by technical issues in the NR. The device in facility G was withdrawn after three months due to charging problems. In facilities with a fixed device, however, consistent access did not translate into consistent use. Facility E had the lowest usage behavior despite presence of a functioning device for about seven months, confirming that access alone is not sufficient to ensure mHealth compliance [26]. Availability of an alternative POC device in Facility E did not fully explain its low usage. Unavailability of transport, poor intervention ownership and limited on-site supervision influenced successful device rotation between facilities B and C. Temporary absence of users, lack of electricity, technical failure, poor supervision and poor technical skills, are commonly reported barriers to implementing diagnostic and decision support systems and may also explain dips in our trend analysis [12,24]. Chaiyachati et al. noted that proper training and continuous technical support did not mitigate low compliance in their study, suggesting the influence of broader multifaceted factors [25].

Consistent with rapidly rising usage in the early weeks of technology introduction in other studies, we observed the “novelty effect” [27]. However, this was not sustained and may explain why initial rates were not maintained. Usage levels could be systematically sustained by applying user-centred designs, iterative feedback processes and ensuring workflow integration [28]. B4M use in facilities A, B and D spiked in phase V after a preceding dip, coinciding with the period when users were informed of intentions to evaluate the project. This manifestation of the Hawthorne effect- people acting as expected when conscious of being observed [29], emphasizes the importance of supervision and monitoring in sustaining mHealth compliance. In order to be effective, supervision must go beyond a checklist approach. A study in Northern Ghana found that health workers only respond positively to supportive supervision [30]. Frimpong et al. add that timing, duration and frequency of monitoring should be taken into account when leveraging the influence of supervision [30].

Women did not undergo their first B4M screening in early pregnancy with continuation till delivery as was expected and more women commenced B4M monitoring in their second and third trimesters. We do not exclude inaccuracies in the estimation of gestational age in explaining this result as health workers sometimes guess Figures [31]. Coverage of 4+ ANC visits with B4M was about 3%, although aggregate data from the three intervention districts show that the percentage of women attending up to four ANC visits is at least 50% [6]. This is likely due to known sociocultural and financial factors which posed a challenge to early monitoring and follow-up with the device [32,33]. Qualitative data from the project confirm that health workers did not systematically use the device, therefore a first B4M visit may actually be a woman’s third or fourth ANC visit [15].

Viability of POC tests have been widely debated along the lines of cost-effectiveness, accuracy and clinical effects, specifically with low-resource settings in mind [34]. Direct and indirect costs associated with ANC attendance are reported barriers to early and sustained visits [35,36]. Even in facilities with laboratories, stock-outs, high costs and long waiting times are barriers to routine antenatal screening [4]. Despite inconsistent use, we anticipate that by eliminating the need for diagnostic referral for basic tests in at least 335 women, decentralised screening helped save money and time. Beneficiaries report that B4M saved time and money, with higher perception of time efficiency in larger facilities [15]. We therefore expect that women with no indications for referral urgency, who attended ANC in facilities with on-site capabilities, may have benefitted from reduced waiting time due to expanded diagnostic options.

The effectiveness of POC testing has been rightly identified as being only as good as the resulting action [37] but we were unable to ascertain if B4M users accepted and acted on the decision recommendations. B4M referral warnings were sometimes repeat recommendations, suggesting that health workers did not adhere to referral recommendations or women did not comply. Detection of risk factors in B4M follow-up visits also emphasises the importance of repeat screening. Although beyond the scope of this paper, it is worth mentioning that implicit trust in the accuracy of diagnostic components extends to the resultant decision support recommendation. Congruence between referral decision and diagnostic test for haemoglobin was better with higher Hb values, while BP screening gave some extremely low or high values (**Figures 5 **and** 6**). The role that analytical or user-dependent errors played in explaining these is uncertain. Nevertheless, the importance of diagnostic validity, technology design and user training already put forward by other researchers requires emphasis [24,26,38]. Caution must be added that B4M diagnostic tests were more conclusive for hypertension and glycosuria than hypertension-in-pregnancy and gestational diabetes respectively, for which additional criteria and tests are mandated.

Overall, the benefits of POC diagnostic and decision support strategies outweigh their limitations [12,24,26]. Continuous training, integration of results with existing health information systems and supportive on-site supervision to ensure correct technique are useful strategies to sustain adoption and usage behavior of mHealth [24,25,37]. Going forward, modifications to the B4M prototype can reduce invalid data and time needed to complete a visit by automating more functions and enabling prompts that flag invalid or missing data. Data synchronization in a secure cloud database can allow instantaneous dashboard analytics, giving immediate feedback to users. By functioning as an invisible eye, active electronic monitoring may simulate the Hawthorne effect indirectly [39]. Although the financial trade-off for incorporating and maintaining solar panels into the device was not feasible under the evaluated prototype, an alternative power source is necessary to reduce reliance on electricity and allow device use for community outreach visits. Lessons learnt from the B4M experience are applicable to similar interventions in other health domains. For example, the prototype can be contextually adapted to support integrated measurement and monitoring of diseases such as HIV, malaria and tuberculosis, as demonstrated by an on-going study in Nigeria [13].

### Limitations

Our analysis was limited by unavailable data due to entry errors and partial screening visits. About 20% of all B4M records had dating errors for visit date or expected date of delivery. The majority of these were from facility F after a glitch resulted in incorrect dates for 302/337 (89.6%) records from this site. Missing data on gestational age affected trimester analysis for 381/940 (40.5%) women. However, we had valid data for at least 90% of all B4M diagnostic results and referral decisions- the core components of the intervention. Poor feedback on referrals and fragmented recordkeeping at health facilities made it challenging to ascertain how many women had eventually been referred and had complied with B4M referral. With the exception of identifying women in high-risk age groups, other socio-demographic data were not collected and device compliance at an individual level was not assessed. Although these narrowed the extent to which the database could be further interrogated to identify factors explaining heterogeneous usage and for whom the device was most beneficial, facility level analysis was possible. While the economic implications of maintaining devices like B4M in low-resource settings is crucial to inform its sustainability, we were unable to present conclusive figures on costing and cost-effectiveness of the device. Finally, the nature and scope of the B4M intervention as a short-term proof of concept in seven health facilities limits transferability of findings to similar contexts within the Ghanaian health system. Nevertheless, findings are relevant for improving the device prototype and we conducted a district level stakeholders (health workers and district administrators) meeting at project end to discuss findings. These are necessary for further implementation research that can provide evidence on the possibility of bringing such an innovation to scale.

## CONCLUSIONS

The B4M pilot suggests that similar interventions may help decrease unnecessary diagnostic referrals for women in rural settings and support continuity of care especially at primary level. Interactions between implementation strategies, technical features, behavioural dispositions, and context influenced usage behaviour and compliance. As many low- and middle-income countries (LMICs) aspire to improve maternal health services while grappling the obstetric transition and the promise of technology, factors underlying why some facilities perform better than others and how these can be overcome in the design and implementation phases require further research. While it may be premature to assess clinical outcomes, the potential to reduce the economic impact of diagnostic referral on pregnant women is worth investigating.
